# Utilization of paediatric isolation facilities in a TB-endemic setting

**DOI:** 10.1186/s13756-015-0078-z

**Published:** 2015-09-21

**Authors:** Angela Dramowski, Mark F. Cotton, Andrew Whitelaw

**Affiliations:** Department of Paediatrics and Child Health, Division of Paediatric Infectious Diseases, Faculty of Medicine and Health Sciences, Stellenbosch University, PO Box 241, Cape Town, 8000 South Africa; Department of Medical Microbiology, Stellenbosch University and the National Health Laboratory Service (NHLS), Cape Town, South Africa

**Keywords:** Paediatrics, Healthcare-associated infection, Nosocomial infection, Infection control, Patient isolation, Transmission-based precautions, Tuberculosis

## Abstract

**Introduction:**

In hospital settings, patient isolation is used to limit transmission of certain pathogens (e.g. *M. tuberculosis* [TB], antibiotic-resistant bacteria and viruses causing respiratory and enteric infection). Data is lacking on utilization of paediatric isolation facilities in low-resource, TB-endemic settings.

**Methods:**

Prospective weekday observation of 18 paediatric isolation rooms at Tygerberg Children’s Hospital, Cape Town, South Africa, was conducted between 1 May 2014 and 31 October 2014 documenting: occupancy rate; indication for isolation; duration of isolation; application of transmission-based precautions and infection prevention (IPC) behaviour of personnel. Potential under-utilization of isolation rooms was determined by cross-referencing isolation room occupancy with laboratory isolates of antibiotic-resistant bacteria, *M. tuberculosis* and selected viral pathogens.

**Results:**

Six percent (335/5906) of hospitalized children were isolated: 78 % (260/335) for IPC purposes. Most IPC-isolated patients had community-acquired infections (213/260; 82 %), including tuberculosis (130/260; 50 %) and suspected viral infections (75/260; 29 %). Children (median age 17 months [IQR 6–50]) spent 4 days (IQR 2–8) in isolation. Isolation occupancy was 66 % (2172/3294 occupied bed days), but varied significantly by month. Laboratory data identified an additional 135 patients warranting isolation with 2054 extra bed-days required. Forty patients with 171 patient days of inappropriate isolation were identified. During 1223 weekday visits to IPC-isolated patient rooms: alcohol-based handrub was available (89 %); transmission-based precautions were appropriately implemented (71 %); and personal protective equipment was provided (74 %). Of 358 observed interactions between paediatric staff and isolated patients, hand hygiene compliance was 65 % and adherence to transmission-based precautions was 58 %.

**Conclusion:**

Patients isolated for TB (under airborne precautions) accounted for more than half of all isolation episodes. Missed opportunities for patient isolation were common but could be reduced by implementation of syndromic isolation. Demand for isolation facilities was seasonal, with projected demand exceeding available isolation beds over winter months.

## Background

Standard and transmission-based precautions (contact, droplet and airborne) [1] are used to interrupt pathogen transmission in healthcare settings. Patient isolation is a key component of these precautions, targeting pathogens such as *M. tuberculosis* (TB), antibiotic-resistant bacteria and certain viruses. Despite widespread implementation of these precautions in high-income countries, the resources to effectively apply patient isolation and transmission-based precaution recommendations are lacking in many low and middle income countries [2, 3].

In paediatric wards, where infectious disease related-admissions predominate, the demand for isolation beds may be ten-fold greater than for hospitalized adults [4]. Not only are hospitalized children more likely to transmit infection, they are also at elevated risk for healthcare-associated infection (HAI) owing to immunological immaturity, underdeveloped mucosal barriers and increased handling by healthcare staff [5]. Studies of isolation facility usage in high-income settings report that 5-17 % of paediatric patients need isolation for IPC purposes, mostly for community-acquired infections (60–75 %) [4, 6–8]. Marked seasonality in demand for paediatric isolation beds (with demand often exceeding supply) is reported even from facilities with more single rooms than cohort beds [4, 6, 7]. The most common precaution type implemented in paediatric studies [5, 8, 9] is contact precautions (80–90 %) followed by droplet precautions [6]. A single study [6] measured correct use of isolation room precaution signage (93 %) and availability of personal protective equipment (100 %). Staff compliance with hand hygiene and transmission-based precautions recommendations in paediatric isolation rooms was not evaluated in these studies.

The impact of absent or limited paediatric isolation facilities in low-middle income settings is unquantified, but undoubtedly promotes infection transmission, along with adverse health system factors like overcrowding and lack of IPC provisions [3]. Data on rates of patient isolation, indications for and utilization patterns of paediatric isolation facilities in Africa is lacking. We evaluated isolation facility utilization and transmission-based precaution implementation at a paediatric referral hospital in a TB-endemic setting in Cape Town, South Africa.

## Methods

### Setting and patient profile

The Tygerberg Children’s Hospital (TCH) in Cape Town, South Africa has 300 paediatric beds within the 1384-bed academic hospital complex. Sick neonates, infants and children (0–14 years) from Cape Town’s Metropole East are hospitalized in 13 neonatal and paediatric wards (including surgical, general medical, sub-specialty wards and intensive care). Admissions reflect a high burden of infectious disease with TB, lower respiratory tract infections and gastroenteritis predominating. HIV prevalence in paediatric inpatients is approximately 15 %; antiretroviral therapy is widely available and improved access to prevention of mother-to-child HIV infection transmission programmes (PMTCT) has reduced national vertical HIV transmission rates to 2.4 % in 2012 [9]. Immunisation coverage rates were 88 % in infants under 12 months of age in 2012 [10].

Patient isolation in our context implies placement of a patient in a single room with application of transmission-based precautions based on clinical indication. Five paediatric wards have 18 single rooms available for patient isolation (9 under negative pressure; only 3 with en-suite bathrooms; none have ante-rooms). This represents 14 % of the available beds on the five wards: 1 room in acute admissions, 3 in paediatric surgery, 2 in general paediatrics, 2 in pulmonology/neurology and 10 in the infectious diseases/gastroenterology ward. The paediatric intensive care unit (PICU) has 10 beds in 2 cohort rooms with no single room or isolation facilities available. PICU patients were eligible for study inclusion if transferred to any of the specified wards.

Each ward’s medical and nursing personnel determine which patients are placed in isolation. Although there is no formal policy guiding isolation room utilization, preference is given to patients requiring airborne precautions e.g. pulmonary TB, measles and varicella. Where possible, children are accompanied by a caregiver (who may also require isolation e.g. for pulmonary TB). Syndromic isolation for suspected viral diseases is infrequently implemented, owing to clinician unfamiliarity with the practice and limited isolation space. In contrast, syndromic isolation for suspected TB is actively practiced, based on compatible symptoms and signs, history of TB contact and/or a suggestive chest radiograph appearance. The hospital’s Unit for Infection Prevention and Control (IPC) conducts laboratory surveillance for selected bacterial “alert” pathogens and makes recommendations for patient isolation on an ad-hoc basis. Infectious patients are usually isolated until discharge (with the exception of meningococcal disease with de-isolation after 24 h of therapy).

### Study design and data collection

Prospective observation of paediatric isolation rooms was conducted on weekdays from 1 May 2014 to 31 October 2014 documenting: occupancy rates; indication for isolation; duration of patient isolation and application of transmission-based precautions using the 2007 CDC patient isolation indications [1]. During weekday visits, observed interactions between personnel and isolated patients were documented including compliance with hand hygiene and transmission-based precautions. Hand hygiene was scored as compliant if all potential opportunities for hand hygiene were followed, or non-compliant if all or some opportunities for hand hygiene were missed. Transmission-based precautions were scored as compliant if all recommendations and appropriate personal protective equipment was applied, or non-compliant if all or some recommendations were not followed. Alcohol handrub and personal protective equipment was scored as available, if the handrub and equipment items needed (based on the appropriate precautions) were supplied at the entrance to the isolation room.

### Estimation of isolation room under-utilization

Two datasets were created: “patient isolation room utilization data” was collected on weekday ward rounds using Microsoft Access 2013 and “patients with pathogens warranting isolation” was extracted from Microbiology and Virology laboratory databases. Pathogens warranting isolation [1] included: multidrug-resistant (MDR) bacteria [11] from any clinical specimen (blood culture, urine, tissue, pus, wound swab and catheter tip) including methicillin-resistant *S. aureus* [MRSA], carbapenem-resistant *A. baumanni* [CRAB], MDR *P. aeruginosa* [MDR PA] and extended spectrum B-lactamase producing Enterobacteriaceae [ESBL]; *M. tuberculosis* both drug-susceptible (DS) and drug-resistant (DR) isolated from any respiratory sample (gastric washing and/or induced sputum) on GeneXpert, smear microscopy or TB culture; and viruses including enteric (rota and adenovirus, hepatitis A virus) and respiratory pathogens (respiratory syncytial virus, adenovirus, human rhinovirus, parainfluenza 1/2/3, influenza A/B and human metapneumovirus) identified by rapid assays, enzyme-linked immunosorbent assays (ELISA) or polymerase chain reaction (PCR) panel testing. All laboratory investigations were taken at the discretion of attending clinicians. Repeated isolates of the same pathogen from one or more sites counted as a single infection episode warranting patient isolation.

The laboratory pathogen dataset was cross-referenced against the patient isolation room utilization dataset. Several measures of isolation room utilization were calculated: missed isolation (patients with pathogens warranting isolation who were not isolated), delayed isolation (patients with late imposition of isolation precautions), inappropriate isolation (no clinical indication for isolation; failure to de-isolate after no pathogens or pathogens not warranting isolation were identified and failure to de-isolate after appropriate therapy) and syndromic isolation requirement (patients with suspected viral infections who were not isolated and had a negative laboratory test results for viral pathogens by day four of hospitalisation). Additional isolation bed days required were calculated by adding the length of stay for each patient who was identified as having a missed indication for IPC-isolation. Additional syndromic bed-days were calculated by adding the number of days from hospital admission to a negative test result for specified viral pathogens for each patient (mean interval was 3.5 days). Additional days of pathogen exposure were calculated as days of missed isolation plus days of delayed isolation.

### Statistical analysis and ethical approval

Descriptive statistical analyses were performed using Stata Statistical Software version 13.0 IC (College Station, TX: StataCorp LP). Median length of stay was compared using the Mann–Whitney test. A p-value below 0.05 was considered statistically significant. Ethical approval and waiver of individual informed consent was obtained from the Human Health Research Ethics committee of Stellenbosch University (S13/09/171).

## Results

Six percent (335/5906) of children admitted during the study period were placed in isolation. Most isolation episodes (260/335; 78 %) were for IPC indications. Community-acquired infections were the predominant reason for isolation (213/260; 82 %), including TB disease (130/260; 50 %) and suspected viral infections (75/260; 29 %) (Table 1). Non-IPC indications for isolation included nursing considerations (post-operative care or provision of total parenteral nutrition), palliation, behavioral or protective isolation. In 11/335 (3 %) episodes, no indication for isolation was identified. Children (median age 17 months; IQR 6–50) were isolated for a median of 4 (IQR 2–8) days. Patients isolated for suspected or confirmed TB stayed longer than patients with other infectious indications for isolation (median of 5 versus 3.5 days; *p =* 0.006). Overall isolation room occupancy was 66 % (2172 occupied/3294 available bed days), but varied significantly by month with peak usage in winter months (June [76 %], July [77 %], August [87 %]).

**Table 1:** Paediatric isolation room utilization

Variable	Total	Percentage	Interquartile range
Discrete patient isolation episodes	335	100	-
Median patient age (months)	17	-	6–50
Median stay in isolation room (days)	4	-	2–8
Indication for isolation			
- infection control (IPC) purposes	260	78	
- nursing care	46	14	-
- palliation/privacy	13	4	
- other^a^	16	4	
Transmission-based precautions^b^ applied	260	100	
- airborne precautions	136	52	-
- droplet precautions	57	22	
- contact precautions	67	26	
	Mean	Minimum	Maximum
Isolation room occupancy rate^c^	2172/3294	225/540	487/558
	(66 %)	(42 %)	(87 %)

IPC = infection prevention and control

^a^other = no obvious reason for isolation (*n =* 11), behavioural isolation (*n =* 3), protective isolation (*n =* 2)

^b^Using the 2007 CDC isolation guidelines[1]

^c^Calculated as the sum of days when isolation rooms (*n =* 18) were occupied divided by [total isolation bed capacity x number of days in the observation period] i.e. 6 months to calculate mean or 1 month to calculate minimum and maximum occupancy rates

TB disease (130/260; 50 %) was the most frequent admission diagnosis in IPC-isolated patients [Table 2]. In 55 children (42 %) microbiological confirmation was obtained; twelve children (9 %) had smear-positive tuberculosis (1–99 acid-fast bacilli per high power field) and 12/45 (26 %) children with culture-positive TB had drug-resistant disease.

**Table 2:** Microbiological isolates^a^ from patients in isolation for IPC purposes

Category	Variable	Total (%)
All suspected TB (*n =* 130)	pulmonary TB	118 (91)
	extra-pulmonary TB^b^	12 (9)
TB smear microscopy (*n =* 130)	not tested (diagnosis confirmed at referral hospital)	10 (8)
	smear-negative	108 (83)
	smear-positive (1–10 AFB/field)	8 (6)
	smear-positive (11–99 AFB/field)	4 (3)
Confirmed TB^c^ (*n =* 55)	TB diagnosis confirmed at referral hospital	10
	GeneXpert positive	43
	TB culture positive	45
Drug-susceptibility profile of culture positive cases (*n =* 45)	Drug-susceptible (DS)	33 (74)
	Multidrug-resistant (MDR)	9 (20)
	Rifampicin mono-resistant (RMR)	2 (4)
	Extensively drug-resistant (XDR)	1 (2)
		Total (number DR)
All ^a^bacterial/fungal isolates cultured from patients in isolation (*n =* 46)	Gram positives	
	*S. aureus* total (methicillin-resistant)	7 (2)
	Other gram positives^d^	4
	Gram negatives: Enterobacteriaceae	
	*K. pneumoniae* (extended-spectrum B-lactamase)	11 (10)
	*E. coli* (extended-spectrum B-lactamase)	5 (1)
	*E. cloacae* (inducible B-lactamase)	4 (2)
	Other Enterobacteriaceae^e^	4
	Gram negatives: Non-fermenters	
	*A. baumanni* (multi-drug resistant)	4 (4)
	*P. aeruginosa* (multi-drug resistant)	5 (3)
	Others	
	*N. meningitidis*	2
	*B. pertussis* (PCR positive)	1
	*C. albicans*	2
Viral pathogens confirmed among patients in isolation (*n =* 51)	Gastrointestinal viruses	
	Hepatitis A	6
	Rotavirus	4
	Enteric adenovirus	2
	Respiratory viruses	
	Respiratory syncytial virus	15
	Adenovirus	12
	Rhinovirus	4
	Parainfluenza virus	2
	Other respiratory viruses^f^	3
	Other	
	Varicella	2
	Rubella	1

^a^Some patients were isolated on clinical suspicion of sepsis or suspicion of a pathogen warranting isolation, but laboratory tests subsequently confirmed a pathogen which did not warrant isolation or transmission-based precautions by CDC guidelines[1] e.g. *Candida albicans*; TB = tuberculosis

^b^Extra-pulmonary TB: disseminated (7), TB lymphadenitis (3), TB meningitis (2)

^c^confirmed TB = geneXpert positive or culture positive for mTB; DR = drug-resistant

^d^Other gram positives: *C. difficile* (1), *S. pyogenes* (1), *S. pneumoniae* (1), *E. faecium* (1)

^e^Other Enterobacteriaceae: *S. typhi* (1), *S. non-typhi* (1), *C. freundii* (1), *P. mirabilis* (1)

Other respiratory viruses^f^: Influenza virus (1), Bocavirus (1), Human metapneumovirus (1)

The remaining 130/260 (50 %) patients isolated for IPC indications included suspected or confirmed: viral respiratory infection (49; 19 %), viral gastrointestinal infection (20; 8 %), varicella or measles (6; 2.5 %), nosocomial sepsis (29; 11 %), skin/soft tissue infection (13; 5 %), hepatitis A (7; 3 %) and meningococcal infection (6; 2.5 %). In some patients (isolated on clinical suspicion of infection), laboratory tests revealed no pathogen or a pathogen not requiring isolation/transmission-based precautions (Table 2).

Cross-referencing of patient isolation and laboratory data identified an additional 135 patients warranting isolation (i.e. missed isolation episodes), with an additional 2054 required isolation bed-days. Of patients with missed isolation episodes, 43 (32 %) had MDR bacteria [1167 extra bed-days], 16 (12 %) had newly-diagnosed, microbiologically confirmed drug-susceptible TB [116 extra bed-days] and 76 (56 %) had viral pathogens [771 extra bed-days] (Table 3).

**Table 3:** Missed opportunities^a^ for patient isolation (May – October 2014)

Pathogen type and species	Missed isolation (Non-isolated patients with pathogens warranting isolation)						
	May	June	July	August	Sept.	Oct.	Study period
^b^MDR bacteria							
Methicillin-resistant *S. aureus* (MRSA)	3	0	0	1	2	1	7
Carbapenem-resistant *A. baumanni* (CRAB)	0	1	0	0	0	2	3
MDR *P. aeruginosa* (MDR PA)	0	0	0	1	2	1	4
Extended spectrum B-lactamase producing (ESBL) Enterobacteriaceae	8	8	5	4	1	3	29
^c^ *M. tuberculosis*							
Drug-susceptible (DS)	2	0	3	3	3	5	16
Drug-resistant (DR)	0	0	0	0	0	0	0
Enteric viruses							
Hepatitis A	3	4	5	7	2	2	23
Rotavirus and/or enteric Adenovirus	0	2	2	1	0	0	5
Respiratory viruses							
Respiratory syncytial virus A/B	9	5	2	2	0	1	19
Adenovirus	2	2	3	3	3	1	14
^d^Other respiratory viruses	0	3	4	2	2	4	15
Missed patient isolation episodes^a^	27	25	24	24	15	20	135
Inappropriate isolation room use (days)^e^	29	77	7	30	27	1	171

^**a**^Missed patient isolation opportunities = patients with pathogens warranting isolation that were not isolated plus days of delay in patients with delayed isolation; multiple laboratory isolates of the same pathogen from 1/more sites was considered a single infection episode warranting patient isolation

^b^Multidrug-resistant (MDR) bacteria isolated from a clinical specimen (blood culture, urine, tissue, pus, wound swab, catheter tip) as per proposed definitions [12] (including methicillin-resistant *S. aureus* [MRSA], carbapenem-resistant *A. baumanni* [CRAB], MDR *P. aeruginosa* [MDR PA] and extended spectrum B-lactamase producing Enterobacteriaceae [ESBL])

^c^ *M. tuberculosis (M.tb)* = any form of drug-susceptible (DS) or drug-resistant (DR) *M.tb* isolated on GeneXpert, smear microscopy or TB culture; Viruses included gastrointestinal and respiratory pathogens identified by rapid assays, ELISA or PCR panels

^d^Other respiratory viruses warranting isolation and transmission-based precautions = human rhinovirus, parainfluenza 1/2/3, Influenza A/B and human metapneumovirus

^**e**^Inappropriate isolation room use = total days when isolation rooms were used for inappropriate purposes i.e. without a clinical indication, failure to de-isolate after laboratory testing identified no pathogens or pathogens that did not warrant isolation

**Fig. 1:**
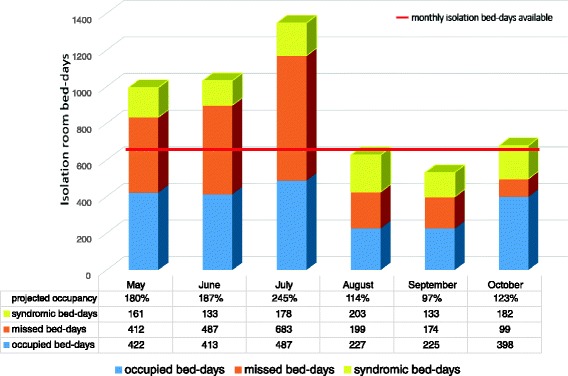
Projected demand for paediatric isolation facilities (May – October 2014). Occupied bed-days = the cumulative daily occupancy of the 18 isolation beds per month; missed bed-days = duration of hospitalization of patients with pathogens warranting isolation that were not isolated plus days of delay in patients with late implementation of isolation precautions; estimated syndromic bed-day requirement = number of patients with clinical suspicion of viral illness but a negative laboratory test/s for viral pathogens (measured as empiric isolation required from day of admission to a negative test result); projected occupancy = sum of occupied bed-days plus missed bed-days plus estimated syndromic days, minus total bed-days of inappropriate use; Bed-days of inappropriate isolation room use (not shown in figure) = total days per period where isolation rooms were used for inappropriate purposes i.e. where there was no clinical indication for isolation or where a patient was not de-isolated after laboratory testing identified no pathogens or pathogens that did not warrant isolation

Of 395 laboratory investigations for viral pathogens warranting isolation: 103 patients with a positive test were identified (26 % yield). Of these 103 patients, 19 (18 %) had been isolated from admission, 8 (8 %) had delayed isolation (mean of 6 days delay) and 76 (74 %) were never isolated. The eight delayed isolation episodes had a combined 51 days of additional pathogen exposure on the ward (including hepatitis A, adenovirus, respiratory syncytial virus, parainfluenza 3 and rhinovirus.) A further 226 patients (with 292 negative laboratory tests for viral pathogens warranting isolation) would have qualified for syndromic isolation on admission. Implementing syndromic isolation for these patients would add an additional 990 days of isolation bed demand, increasing the months where demand would outstrip bed availability from 3 to 5 months in the study period (Fig. 1).

Forty patients with 171 patient days of inappropriate isolation were identified (29 days with no apparent reason for isolation and 142 days where IPC isolation was unwarranted i.e. no pathogens were isolated by day 4 of admission). Overall inappropriate isolation days accounted for 5 % of the available bed days (171/3294). Although total projected isolation bed-days reflected a deficit of 761 days (or 123 % projected occupancy) for missed isolation episodes, the deficit was confined to the first 3 months of the study period during the winter season (Fig. 1). The percentage of paediatric admissions requiring isolation increases from 6 % to 8 % when patients with missed isolation episodes are included and to 12 % when including missed isolation and children investigated for viral pathogens.

During 1223 weekday visits to isolation rooms used for IPC: alcohol-based handrub was generally available (89 %); transmission-based precautions were appropriately implemented (71 %) and personal protective equipment was provided (74 %). Of 358 observed interactions between personnel and isolated patients, hand hygiene compliance was 65 % and adherence to transmission-based precautions was 58 %.

## Discussion

In our study of paediatric isolation room utilization, only 6 % of hospitalized children were placed in isolation. This contrasts with reported isolation rates of 14–17 % in high-income countries, where more single rooms and favourable staffing ratios facilitate greater use of isolation precautions. However, even in high income countries, isolation facilities may be under-utilised. Failure to implement syndromic isolation for suspected viral infections may also explain our institution’s lower isolation rates, supported by the finding of many missed isolation opportunities (particularly among children investigated for viral respiratory and enteric infections). Another explanation for our apparently “low” isolation room occupancy and many “missed” episodes, may be a mismatch between theoretical availability of isolation beds and actual availability of beds. At times of peak isolation demand, even with overall 66 % occupancy, clinicians may struggle to find an open isolation bed at the time that the patient requires it.

In keeping with published reports, community-acquired infections predominated, although TB was the most frequent diagnosis among isolated patients at our institution. Consequently airborne precautions were the predominant precaution type implemented, in stark contrast to studies from high-income settings [4, 6–8]. Since our institution is a referral centre for complicated and/or drug-resistant TB, the findings may over-estimate requirement for airborne isolation facilities in other low-middle income paediatric settings. However, South African TB incidence rates are among the highest worldwide (at 860 per 100 000 population) [12] and therefore a strong argument could be made for provision of airborne isolation at all inpatient facilities.

Paediatric TB is often considered low-risk for nosocomial transmission, but our cohort of 130 patients had substantial rates of smear-positive and/or drug-resistant disease. Children with TB also stayed significantly longer (increasing TB exposure time on the wards), despite expedited transfer to a regional TB treatment facility. Another consideration in TB-endemic settings is the presence of undiagnosed or recently diagnosed pulmonary TB in caregivers accompanying hospitalized children, with several documented instances of nosocomial TB transmission [13–17]. Paediatric wards in TB-endemic settings should implement routine symptom screening of adult caregivers and make allowance for additional isolation space for infectious adults, whenever possible.

Most isolation episodes were for IPC purposes, although nursing considerations also influenced isolation room use. Five percent of isolation bed-days were inappropriately used, highlighting the need for a written guideline on isolation room usage and better staff education about indications for isolation. More importantly, at least 135 opportunities for patient isolation were missed (mostly for MDR bacterial and viral infections). Notably, there were few missed isolation episodes for TB, possibly owing to heightened awareness of isolation recommendations for TB among clinicians.

If patients with missed isolation episodes had been appropriately isolated, an overall shortage of 761 isolation bed-days would have been experienced during the study period (i.e. 123 % projected occupancy). However, missed isolation episodes also showed seasonal fluctuation with greatest projected demand over winter (peaking at 208 % or a deficit of 608 bed-days in June 2014). The true need for isolation beds (actual usage, missed episodes and syndromic episodes) is underestimated by the laboratory cross-referencing methodology used, which only identifies children who had appropriate laboratory investigation/s requested and whose specimens had a positive yield.

In contrast to a Canadian study [6] with almost universally correct use of precaution signage and reliable availability of personal protective equipment (PPE), our rates of 71 % and 74 % respectively were lower. Ongoing in-service training of staff and a standardized isolation policy should improve these rates. Encouragingly, alcohol-based handrub was consistently available (89 %), although hand hygiene compliance was lower at 65 %. Concurrently collected hand hygiene audit data on the same wards using the “secret shoppers” method, recorded compliance rates of only 41 % (personal communication, Marina Aucamp, Professional Nurse). The Hawthorne effect (where individuals modify or improve behaviour in response to awareness of being observed) may have resulted in our “higher” compliance rate, but these rates are concerning given that staff knew they were handling infectious patients.

Paediatric staff at our institution [18] self-report high rates of adherence (80 %) to transmission-based precaution recommendations, but observed compliance was low (58 %). Although appropriate PPE was sometimes unavailable, when it was reliably supplied staff either took no precautions or applied only certain aspects. The additional time and effort required to nurse patients under isolation precautions is well-described [19] and may discourage compliance, together with staff shortages and a lack of education about isolation recommendations.

Limitations of this study include: the short duration (6-months) with inability to estimate annual isolation bed demand and evaluate impact of seasonal disease fluctuations; the tertiary hospital setting (with 18 isolation beds) which differs from most “open-plan” paediatric wards in low-middle income settings; the observation process (Hawthorne effect) which may have increased staff compliance; the method for identification of missed isolation episodes based on laboratory isolation of specific pathogens, potentially underestimating the true demand for isolation (particularly if syndromic isolation were to be implemented).

Notwithstanding these limitations, this study of paediatric isolation utilization has relevance for our own institution and paediatric wards in other low-resource, TB-endemic settings. To accommodate for peaks in demand and facilitate implementation of syndromic isolation, we propose that new paediatric facilities in our setting have a minimum of 40 % of available beds as single or double cohort isolation rooms. Provision for airborne precautions (needed for half of all isolation episodes in this setting), should be prioritised in new facilities and for renovations of existing paediatric wards in TB-endemic settings. Sufficient provision should be made for administrative/office space during design of new wards or facilities, to avoid isolation rooms being inappropriately utilized for non-clinical activities. Syndromic isolation for suspected infection should be implemented (with prompt de-isolation after negative laboratory tests), to reduce risk of infection transmission on children’s wards in low-resource settings. To ensure rational and co-ordinated use of isolation beds in our 300-bedded children’s hospital, nursing or infection control practitioners could be trained as isolation bed managers. Staff education, written policies and active management of scarce paediatric isolation resources are required.

## Conclusion

Most children admitted to isolation facilities in our TB-endemic setting require airborne precautions. Missed opportunities for patient isolation are common, and could be reduced by implementation of syndromic isolation. Actual and projected demand for isolation facilities exceeds available capacity over the winter season.

## Abbreviations

CRAB: Carbapenem-resistant *Acinetobacter baumanni*
DS: Drug-susceptible
DR: Drug-resistant
ESBL: Extended spectrum B-lactamase producing Enterobacteriaceae
HAI: Healthcare-associated infection
HIV: Human immunodeficiency virus
IPC: Infection prevention and control
IQR: Interquartile range
LMIC: Low-middle income country
MDR: Multidrug-resistant
MDR PA: Multidrug-resistant *Pseudomonas aeruginosa*
MRSA: Methicillin-resistant *Staphylococcus aureus*
M.tb: *Mycobacterium tuberculosis*
PMTCT: Prevention of mother to child transmission of HIV
PICU: Paediatric intensive care unit
PPE: Personal protective equipment
TB: Tuberculosis
TCH: Tygerberg Childrens’ Hospital
